# Supplementary material for: Genetic Diversity of Selected High-Risk HPV Types Prevalent in Africa and Not Covered by Current Vaccines: A Pooled Sequence Data Analysis
Source: Int J Mol Sci. 2025 Nov 15;26(22):11056. doi: 10.3390/ijms262211056 (PMC12652174; doi:10.3390/ijms262211056)
Supplement: Supplementary file 1 [file ijms-26-11056-s001.zip › Supplementary Accession list for variant calling.pdf]

Accession numbers considered for variant calling

E5	E6	E7	L1
OP971046.1	OP971046.1	KP313774.1	AY177679.1
OP971062.1	OP971062.1	KP313775.1	OP971046.1
OP971067.1	OP971067.1	OP971046.1	OP971062.1
OP971070.1	OP971070.1	OP971062.1	OP971067.1
OP971080.1	OP971080.1	OP971067.1	OP971070.1
OP711983.1	OP711986.1	OP971070.1	OP971080.1
OP711986.1	OP712015.1	OP971080.1	OP711983.1
OP712021.1	OP712021.1	OP711986.1	OP711986.1
OP712041.1	OP712041.1	OP712015.1	OP712015.1
OP712079.1	OP712079.1	OP712021.1	OP712021.1
OP712097.1	OP712097.1	OP712041.1	OP712041.1
MW836821.1	KX545354.1	OP712079.1	OP712079.1
OP971042.1	KX545359.1	OP712097.1	OP712097.1
OP971048.1	MW836821.1	MW836821.1	MW836821.1
OP971083.1	OP971042.1	OP971042.1	OP971042.1
OP711995.1	OP971048.1	OP971048.1	OP971048.1
OP712011.1	OP971083.1	OP971083.1	OP971083.1
OP712029.1	OP711995.1	OP711995.1	OP711995.1
OP712056.1	OP712011.1	OP712011.1	OP712008.1
OP712070.1	OP712029.1	OP712029.1	OP712011.1
OP712112.1	OP712070.1	OP712070.1	OP712029.1
MT217297.1	OP712112.1	OP712112.1	OP712056.1
MT217304.1	MT217297.1	MT217297.1	OP712070.1
MT217348.1	MT217304.1	MT217304.1	OP712112.1
MT217349.1	MT217348.1	MT217348.1	MT217297.1
MT217355.1	MT217349.1	MT217349.1	MT217304.1
MT217305.1	MT217355.1	MT217355.1	MT217348.1
MT217306.1	OR877117.1	MT217305.1	MT217349.1
MT217307.1	OR877118.1	MT217306.1	MT217350.1
MT217317.1	OR877119.1	MT217307.1	MT217355.1
MT217335.1	OR877120.1	MT217317.1	MT217305.1
MT217336.1	OR877121.1	MT217335.1	MT217306.1
MT217337.1	OR877122.1	MT217336.1	MT217307.1
MT217338.1	OR877123.1	MT217337.1	MT217317.1
MT217339.1	OR877124.1	MT217338.1	MT217335.1
MT217340.1	MT217305.1	MT217339.1	MT217336.1
MT217341.1	MT217307.1	MT217340.1	MT217337.1
MT217342.1	MT217317.1	MT217341.1	MT217338.1
MT217343.1	MT217335.1	MT217342.1	MT217339.1
MT217344.1	MT217336.1	MT217343.1	MT217340.1
MT217345.1	MT217337.1	MT217344.1	MT217341.1
MT217346.1	MT217339.1	MT217345.1	MT217342.1
MT217347.1	MT217340.1	MT217346.1	MT217343.1
MT217351.1	MT217341.1	MT217347.1	MT217344.1
MT217352.1	MT217342.1	MT217351.1	MT217345.1

MT217353.1	MT217343.1	MT217353.1	MT217346.1
MT217384.1	MT217344.1	MT217384.1	MT217347.1
MT217407.1	MT217345.1	MT217407.1	MT217351.1
MT217408.1	MT217346.1	MT217408.1	MT217352.1
MT217436.1	MT217347.1	MT217436.1	MT217353.1
MT217437.1	MT217351.1	MT217437.1	MT217384.1
MT217438.1	MT217353.1	MT217438.1	MT217407.1
MT217439.1	MT217384.1	MT217439.1	MT217408.1
MT217183.1	MT217407.1	MT217181.1	MT217436.1
MT217184.1	MT217408.1	MT217182.1	MT217437.1
MT217185.1	MT217436.1	MT217183.1	MT217438.1
MT217186.1	MT217437.1	MT217184.1	MT217439.1
MT217187.1	MT217438.1	MT217185.1	MT217181.1
MT217188.1	MT217439.1	MT217186.1	MT217182.1
MT217189.1	MT217181.1	MT217187.1	MT217183.1
MT217190.1	MT217182.1	MT217188.1	MT217184.1
MT217385.1	MT217183.1	MT217189.1	MT217185.1
MT217386.1	MT217184.1	MT217190.1	MT217186.1
MT217387.1	MT217185.1	MT217385.1	MT217187.1
MT217400.1	MT217186.1	MT217386.1	MT217188.1
MT217325.1	MT217187.1	MT217387.1	MT217189.1
MT217334.1	MT217188.1	MT217400.1	MT217190.1
MT217301.1	MT217189.1	MT217325.1	MT217385.1
MT217302.1	MT217385.1	MT217334.1	MT217386.1
MT217303.1	MT217386.1	MT217302.1	MT217387.1
MT217320.1	MT217387.1	MT217320.1	MT217400.1
MT217321.1	MT217400.1	MT217321.1	MT217325.1
MT217330.1	MT217325.1	MT217330.1	MT217334.1
MT217358.1	MT217334.1	MT217358.1	MT217301.1
MT217359.1	KX545355.1	MT217359.1	MT217302.1
MT217360.1	MT217320.1	MT217360.1	MT217320.1
MT217456.1	MT217321.1	MT217456.1	MT217321.1
MT217457.1	MT217330.1	MT217457.1	MT217330.1
MT217458.1	MT217358.1	MT217458.1	MT217358.1
MT217460.1	MT217359.1	MT217459.1	MT217359.1
MT217461.1	MT217360.1	MT217460.1	MT217360.1
MT217462.1	MT217456.1	MT217461.1	MT217456.1
MT217463.1	MT217457.1	MT217462.1	MT217457.1
MT217464.1	MT217459.1	MT217463.1	MT217458.1
MT217465.1	MT217460.1	MT217464.1	MT217459.1
MT217466.1	MT217461.1	MT217465.1	MT217460.1
MT217467.1	MT217462.1	MT217466.1	MT217461.1
MT217468.1	MT217464.1	MT217467.1	MT217462.1
MT217469.1	MT217465.1	MT217468.1	MT217463.1
MT217470.1	MT217466.1	MT217469.1	MT217464.1
MT217471.1	MT217467.1	MT217470.1	MT217465.1
MT217472.1	MT217468.1	MT217471.1	MT217466.1
MT217473.1	MT217469.1	MT217472.1	MT217467.1

MT217474.1	MT217470.1	MT217473.1	MT217468.1
MT217475.1	MT217471.1	MT217474.1	MT217469.1
JX129488.1	MT217472.1	MT217475.1	MT217470.1
MT217191.1	MT217473.1	JX129488.1	MT217471.1
MT217192.1	MT217474.1	MT217191.1	MT217472.1
MT217194.1	MT217475.1	MT217192.1	MT217473.1
MT217197.1	JX129488.1	MT217193.1	MT217474.1
MT217198.1	MT217191.1	MT217194.1	MT217475.1
MT217271.1	MT217192.1	MT217195.1	JX129488.1
MT217272.1	MT217193.1	MT217196.1	MT217191.1
OP971032.1	MT217194.1	MT217197.1	MT217192.1
OP971034.1	MT217195.1	MT217198.1	MT217193.1
OP971043.1	MT217196.1	MT217199.1	MT217194.1
OP971054.1	MT217197.1	MT217271.1	MT217195.1
OP971063.1	MT217198.1	MT217272.1	MT217196.1
OP971100.1	MT217199.1	OP971032.1	MT217197.1
MT217200.1	MT217271.1	OP971034.1	MT217198.1
MT217201.1	MT217272.1	OP971043.1	MT217199.1
MT217202.1	OP971032.1	OP971054.1	MT217271.1
MT217203.1	OP971034.1	OP971063.1	MT217272.1
MT217204.1	OP971043.1	OP971100.1	OP971032.1
MT217205.1	OP971054.1	MT217200.1	OP971034.1
MT217206.1	OP971063.1	MT217201.1	OP971043.1
MT217207.1	OP971100.1	MT217202.1	OP971054.1
MT217208.1	MT217200.1	MT217203.1	OP971063.1
MT217209.1	MT217201.1	MT217204.1	OP971100.1
MT217210.1	MT217202.1	MT217205.1	MT217200.1
MT217211.1	MT217204.1	MT217206.1	MT217201.1
MT217212.1	MT217205.1	MT217207.1	MT217202.1
MT217213.1	MT217206.1	MT217208.1	MT217203.1
MT217214.1	MT217207.1	MT217209.1	MT217204.1
MT217215.1	MT217208.1	MT217210.1	MT217205.1
MT217216.1	MT217209.1	MT217211.1	MT217206.1
MT217217.1	MT217210.1	MT217212.1	MT217207.1
MT217218.1	MT217211.1	MT217213.1	MT217208.1
MT217219.1	MT217212.1	MT217214.1	MT217209.1
MT217220.1	MT217213.1	MT217215.1	MT217210.1
MT217221.1	MT217214.1	MT217216.1	MT217211.1
MT217222.1	MT217215.1	MT217217.1	MT217212.1
MT217223.1	MT217216.1	MT217218.1	MT217213.1
MT217224.1	MT217217.1	MT217219.1	MT217214.1
MT217225.1	MT217218.1	MT217220.1	MT217215.1
MT217226.1	MT217219.1	MT217221.1	MT217216.1
MT217227.1	MT217220.1	MT217222.1	MT217217.1
MT217228.1	MT217221.1	MT217223.1	MT217218.1
MT217229.1	MT217222.1	MT217224.1	MT217219.1
MT217230.1	MT217223.1	MT217225.1	MT217220.1
MT217231.1	MT217224.1	MT217226.1	MT217221.1

MT217232.1	MT217225.1	MT217227.1	MT217222.1
MT217233.1	MT217226.1	MT217228.1	MT217223.1
MT217234.1	MT217227.1	MT217229.1	MT217224.1
MT217235.1	MT217228.1	MT217230.1	MT217225.1
MT217236.1	MT217229.1	MT217231.1	MT217226.1
MT217237.1	MT217230.1	MT217232.1	MT217227.1
MT217238.1	MT217231.1	MT217233.1	MT217228.1
MT217239.1	MT217232.1	MT217234.1	MT217229.1
MT217240.1	MT217233.1	MT217235.1	MT217230.1
MT217241.1	MT217234.1	MT217236.1	MT217231.1
MT217242.1	MT217235.1	MT217237.1	MT217232.1
MT217243.1	MT217236.1	MT217238.1	MT217233.1
MT217244.1	MT217237.1	MT217239.1	MT217234.1
MT217245.1	MT217238.1	MT217240.1	MT217235.1
MT217246.1	MT217240.1	MT217241.1	MT217236.1
MT217247.1	MT217241.1	MT217242.1	MT217237.1
MT217248.1	MT217242.1	MT217243.1	MT217238.1
MT217274.1	MT217244.1	MT217244.1	MT217240.1
MT217275.1	MT217245.1	MT217245.1	MT217241.1
MT217277.1	MT217246.1	MT217246.1	MT217242.1
MT217278.1	MT217247.1	MT217247.1	MT217243.1
MT217279.1	MT217274.1	MT217248.1	MT217244.1
MT217280.1	MT217275.1	MT217274.1	MT217245.1
MT217281.1	MT217277.1	MT217275.1	MT217246.1
MT217282.1	MT217278.1	MT217276.1	MT217247.1
MT217283.1	MT217280.1	MT217277.1	MT217248.1
MT217284.1	MT217281.1	MT217278.1	MT217274.1
MT217285.1	MT217283.1	MT217279.1	MT217275.1
MT217287.1	MT217284.1	MT217280.1	MT217276.1
MT217288.1	MT217285.1	MT217281.1	MT217277.1
MT217289.1	MT217287.1	MT217283.1	MT217278.1
MT217290.1	MT217289.1	MT217284.1	MT217279.1
MT217291.1	MT217290.1	MT217285.1	MT217280.1
MT217292.1	MT217291.1	MT217287.1	MT217281.1
MT217356.1	MT217292.1	MT217288.1	MT217282.1
MT217364.1	MT217356.1	MT217289.1	MT217283.1
MT217365.1	MT217364.1	MT217290.1	MT217284.1
MT217366.1	MT217365.1	MT217291.1	MT217285.1
MT217367.1	MT217366.1	MT217292.1	MT217287.1
MT217368.1	MT217370.1	MT217356.1	MT217288.1
MT217369.1	MT217371.1	MT217364.1	MT217289.1
MT217370.1	MT217372.1	MT217365.1	MT217290.1
MT217371.1	MT217402.1	MT217366.1	MT217291.1
MT217372.1	MT217403.1	MT217367.1	MT217292.1
MT217402.1	MT217404.1	MT217368.1	MT217364.1
MT217403.1	MT217405.1	MT217369.1	MT217365.1
MT217404.1	MT217406.1	MT217370.1	MT217366.1
MT217405.1	MT217430.1	MT217371.1	MT217367.1

MT217406.1	MT217431.1	MT217372.1	MT217368.1
MT217430.1	MT217432.1	MT217402.1	MT217369.1
MT217431.1	MT217433.1	MT217403.1	MT217370.1
MT217432.1	MT217434.1	MT217404.1	MT217371.1
MT217433.1	MT217435.1	MT217405.1	MT217372.1
MT217434.1	MT217497.1	MT217406.1	MT217402.1
MT217435.1	MT217498.1	MT217430.1	MT217403.1
MT217497.1	MT217499.1	MT217431.1	MT217404.1
MT217498.1	MT217500.1	MT217432.1	MT217405.1
MT217499.1	MT217501.1	MT217433.1	MT217406.1
MT217500.1	OP712025.1	MT217434.1	MT217430.1
MT217501.1	OP712067.1	MT217435.1	MT217431.1
OP712025.1	MN829875.1	MT217497.1	MT217432.1
OP712067.1	MN829876.1	MT217498.1	MT217433.1
MN829875.1	MN829877.1	MT217499.1	MT217434.1
MN829876.1	MN829878.1	MT217500.1	MT217435.1
MN829877.1	MN829879.1	MT217501.1	OP712025.1
MN829878.1	MN829880.1	MT221244.1	OP712067.1
MN829879.1	MN829881.1	MT221245.1	MN829875.1
MN829880.1	MN829882.1	OP712025.1	MN829876.1
MN829882.1	OP971008.1	OP712067.1	MN829877.1
OP711968.1	OP971014.1	MN829875.1	MN829878.1
OP711975.1	OP971038.1	MN829876.1	MN829879.1
OP711980.1	OP971092.1	MN829877.1	MN829880.1
OP712003.1	OP711960.1	MN829878.1	MN829881.1
OP712007.1	OP711988.1	MN829879.1	MN829882.1
OP712028.1	OP711998.1	MN829880.1	OP971008.1
OP712050.1	OP712001.1	MN829881.1	OP971014.1
OP712071.1	OP712026.1	MN829882.1	OP971038.1
OP712076.1	OP712040.1	OP971008.1	OP971092.1
OP712104.1	OP712057.1	OP971014.1	OP711960.1
	OP712096.1	OP971038.1	OP711974.1
	OP971017.1	OP971092.1	OP711988.1
	OP971020.1	OP711960.1	OP711998.1
	OP971047.1	OP711988.1	OP712001.1
	OP971068.1	OP711998.1	OP712026.1
	OP711967.1	OP712001.1	OP712040.1
	OP712016.1	OP712026.1	OP712057.1
	OP712035.1	OP712040.1	OP712096.1
	OP712049.1	OP712057.1	OP971017.1
	OP712058.1	OP712096.1	OP971020.1
	OP712089.1	OP971017.1	OP971047.1
	OP712103.1	OP971020.1	OP971068.1
	OP711968.1	OP971047.1	OP711967.1
	OP711975.1	OP971068.1	OP712016.1
	OP711980.1	OP711967.1	OP712035.1
	OP712003.1	OP712016.1	OP712049.1
	OP712007.1	OP712035.1	OP712058.1

OP712028.1	OP712049.1	OP712089.1
OP712050.1	OP712058.1	OP712103.1
OP712071.1	OP712089.1	OP711968.1
OP712076.1	OP712103.1	OP711975.1
OP712104.1	OP711968.1	OP711980.1
	OP711975.1	OP712003.1
	OP711980.1	OP712007.1
	OP712003.1	OP712028.1
	OP712007.1	OP712050.1
	OP712028.1	OP712071.1
	OP712050.1	OP712076.1
	OP712071.1	OP712104.1
	OP712076.1	
	OP712104.1	
