# Supplementary material for: Genetic Diversity of Selected High-Risk HPV Types Prevalent in Africa and Not Covered by Current Vaccines: A Pooled Sequence Data Analysis
Source: Int J Mol Sci. 2025 Nov 15;26(22):11056. doi: 10.3390/ijms262211056 (PMC12652174; doi:10.3390/ijms262211056)
Supplement: Supplementary file 1 [file ijms-26-11056-s001.zip › Supplementary material_Phylogenetic analysis.pdf]

1. *Phylogenetic analyses*

HPV16

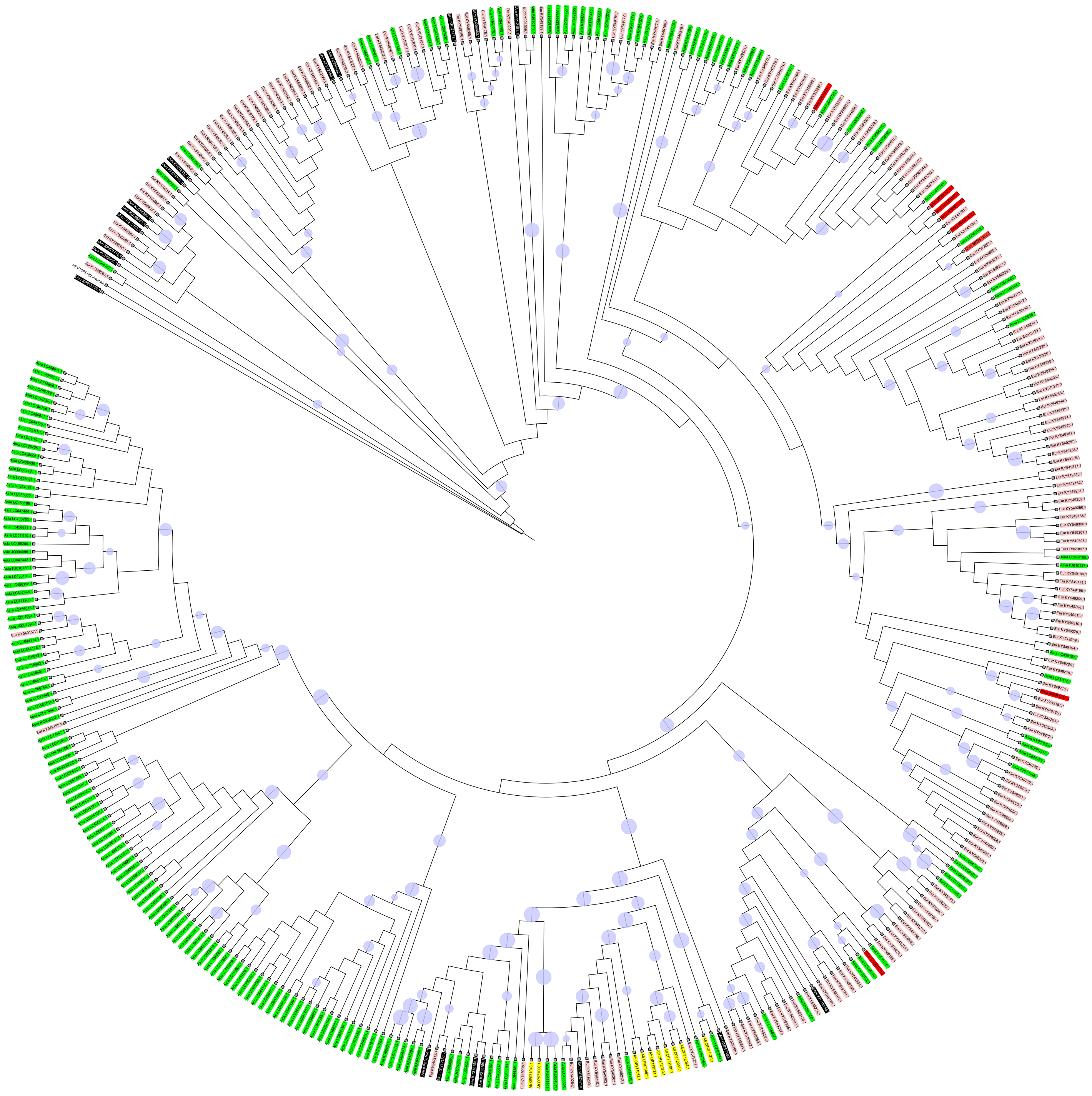

HPV18

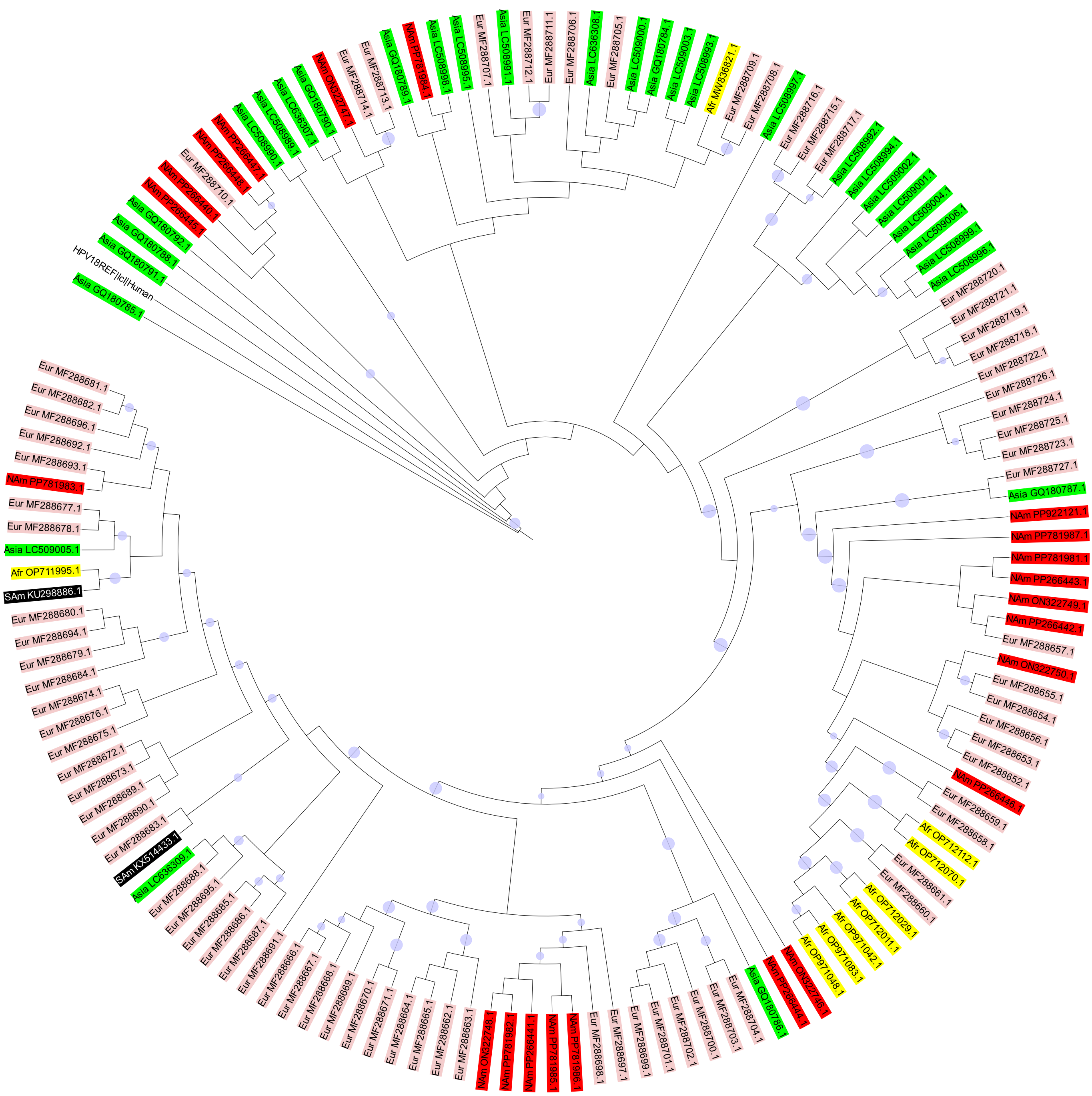

Asia GQ180785.1
HPV18REF|lcl|Human
Asia GQ180791.1
Asia GQ180788.1
Asia GQ180792.1
NAm PP266445.1
NAm PP266440.1
Eur MF288710.1
NAm PP266448.1
NAm PP266447.1
Asia LC508990.1
Asia LC508989.1
Asia LC636307.1
Asia GQ180790.1
NAm ON322747.1
Eur MF288714.1
Eur MF288713.1
Asia GQ180789.1
NAm PP781984.1
Asia LC508998.1
Asia LC508995.1
Eur MF288707.1
Asia LC508991.1
Eur MF288712.1
Eur MF288711.1
Eur MF288706.1
Asia LC636308.1
Eur MF288705.1
Asia LC509000.1
Asia GQ180784.1
Asia LC509003.1
Asia LC508993.1
Afr MW836821.1
Eur MF288709.1
Eur MF288708.1
Asia LC508997.1
Eur MF288716.1
Eur MF288715.1
Eur MF288717.1
Asia LC508992.1
Asia LC508994.1
Asia LC509002.1
Asia LC509001.1
Asia LC509004.1
Asia LC509006.1
Asia LC508999.1
Asia LC508996.1
Eur MF288720.1
Eur MF288721.1
Eur MF288719.1
Eur MF288718.1
Eur MF288722.1
Eur MF288726.1
Eur MF288724.1
Eur MF288725.1
Eur MF288723.1
Eur MF288727.1
Asia GQ180787.1
NAm PP922121.1
NAm PP781987.1
NAm PP781981.1
NAm PP266443.1
NAm ON322749.1
NAm PP266442.1
Eur MF288657.1
NAm ON322750.1
Eur MF288655.1
Eur MF288654.1
Eur MF288656.1
Eur MF288653.1
Eur MF288652.1
NAm PP266446.1
Eur MF288659.1
Eur MF288658.1
Afr OP712112.1
Afr OP712070.1
Eur MF288661.1
Eur MF288660.1
Afr OP712029.1
Afr OP712011.1
Afr OP971042.1
Afr OP971083.1
Afr OP971048.1
NAm ON322746.1
NAm PP266444.1
Asia GQ180786.1
Eur MF288704.1
Eur MF288703.1
Eur MF288700.1
Eur MF288702.1
Eur MF288701.1
Eur MF288699.1
Eur MF288697.1
Eur MF288698.1
NAm PP781986.1
NAm PP781985.1
NAm PP266441.1
NAm PP781982.1
NAm ON322748.1
Eur MF288663.1
Eur MF288662.1
Eur MF288665.1
Eur MF288664.1
Eur MF288671.1
Eur MF288670.1
Eur MF288669.1
Eur MF288668.1
Eur MF288667.1
Eur MF288666.1
Eur MF288691.1
Eur MF288687.1
Eur MF288686.1
Eur MF288685.1
Eur MF288695.1
Eur MF288688.1
Asia LC636309.1
SAm KX514433.1
Eur MF288683.1
Eur MF288690.1
Eur MF288689.1
Eur MF288673.1
Eur MF288672.1
Eur MF288675.1
Eur MF288676.1
Eur MF288674.1
Eur MF288684.1
Eur MF288679.1
Eur MF288694.1
Eur MF288680.1
SAm KU298886.1
Afr OP711995.1
Asia LC509005.1
Eur MF288678.1
Eur MF288677.1
NAm PP781983.1
Eur MF288693.1
Eur MF288692.1
Eur MF288696.1
Eur MF288682.1
Eur MF288681.1

HPV35

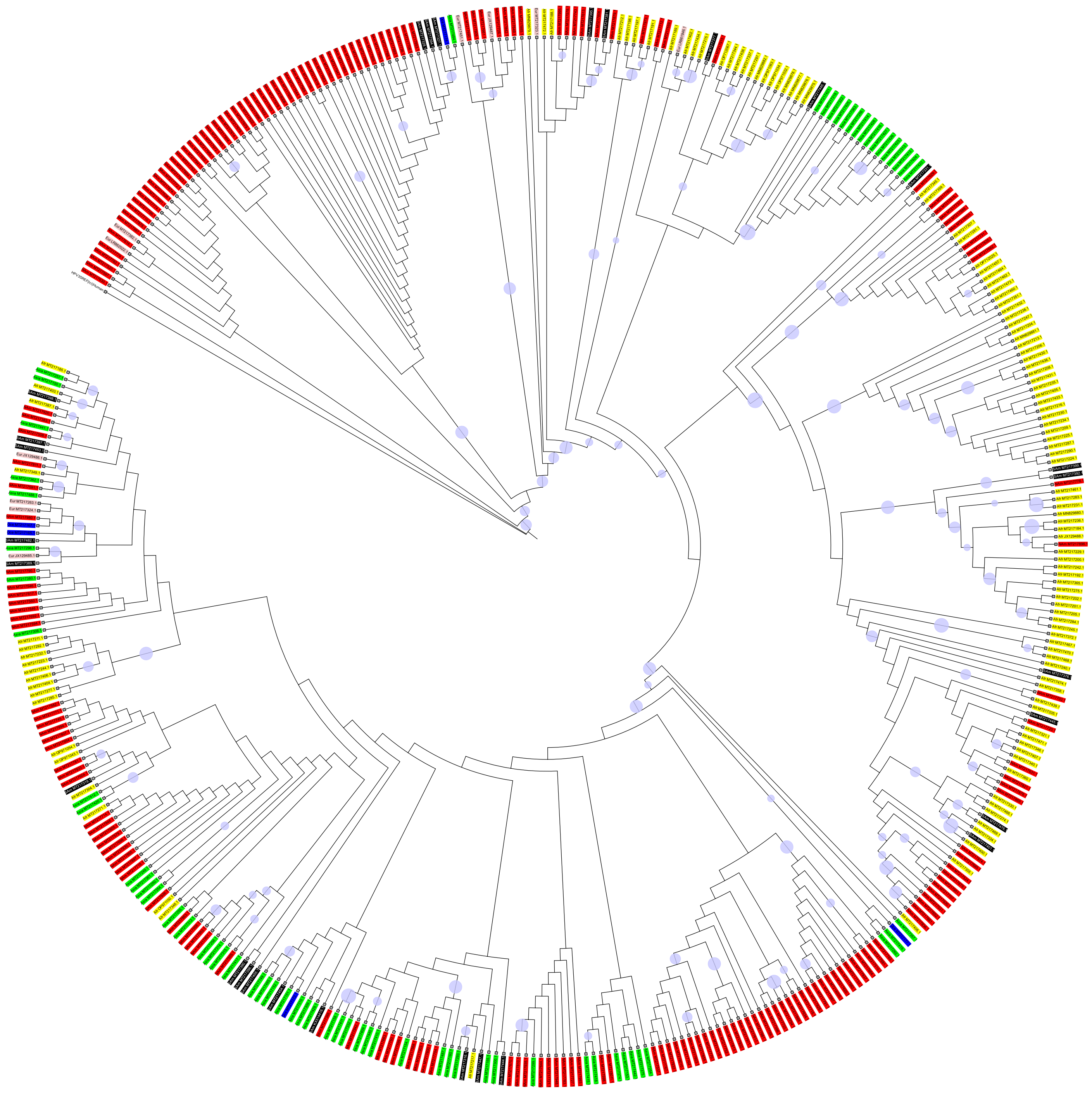

HPV51

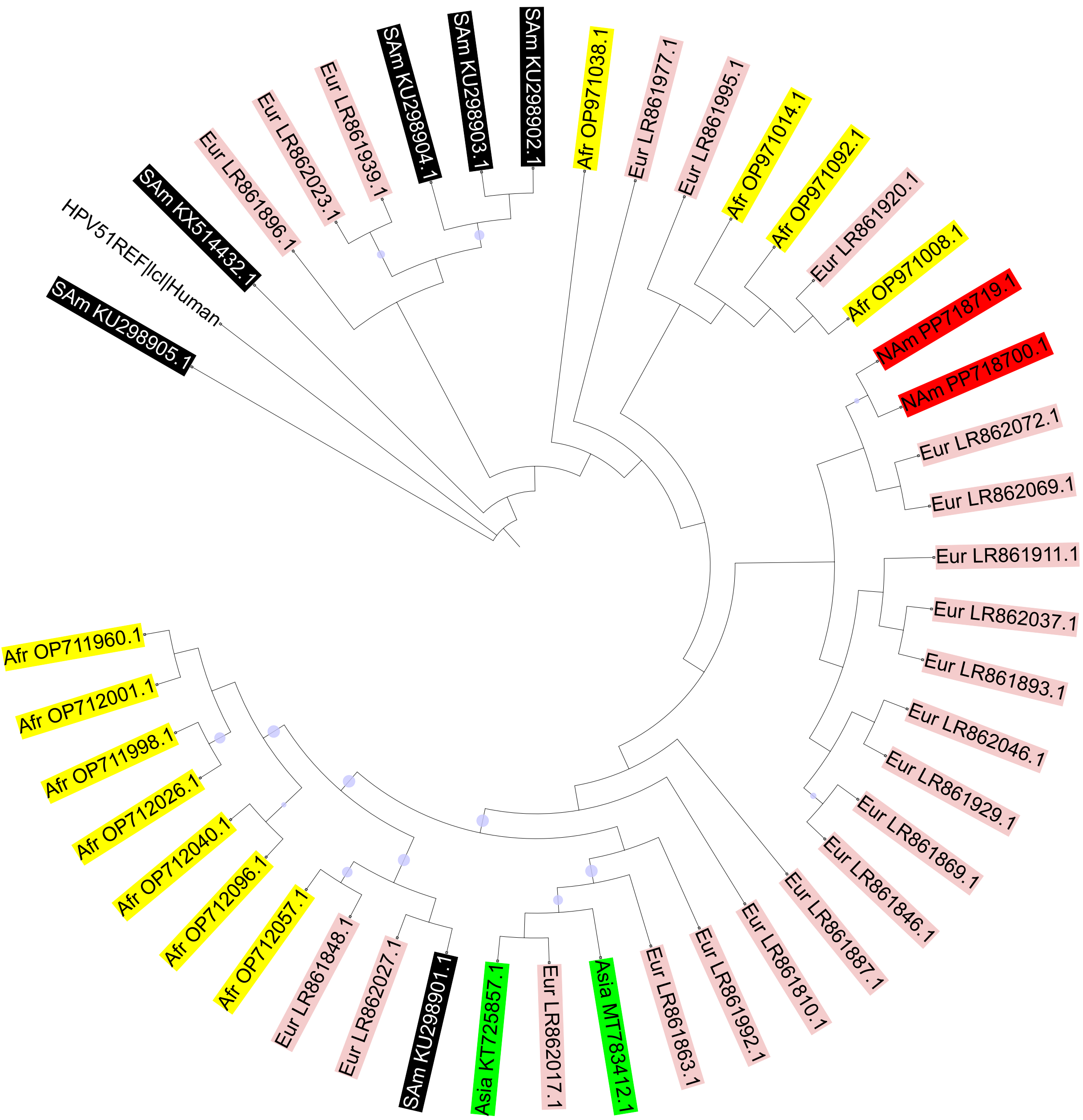
SAm KU298905.1
HPV51REF|lcl|Human
SAm KX514432.1
Eur LR861896.1
Eur LR862023.1
Eur LR861939.1
SAm KU298904.1
SAm KU298903.1
SAm KU298902.1
Afr OP971038.1
Eur LR861977.1
Eur LR861995.1
Afr OP971014.1
Afr OP971092.1
Eur LR861920.1
Afr OP971008.1
NAm PP718719.1
NAm PP718700.1
Eur LR862072.1
Eur LR862069.1
Eur LR861911.1
Eur LR862037.1
Eur LR861893.1
Eur LR862046.1
Eur LR861929.1
Eur LR861869.1
Eur LR861846.1
Eur LR861887.1
Eur LR861810.1
Eur LR861992.1
Eur LR861863.1
Asia MT783412.1
Eur LR862017.1
Asia KT725857.1
SAm KU298901.1
Eur LR862027.1
Eur LR861848.1
Afr OP712057.1
Afr OP712096.1
Afr OP712040.1
Afr OP712026.1
Afr OP711998.1
Afr OP712001.1
Afr OP711960.1

HPV56

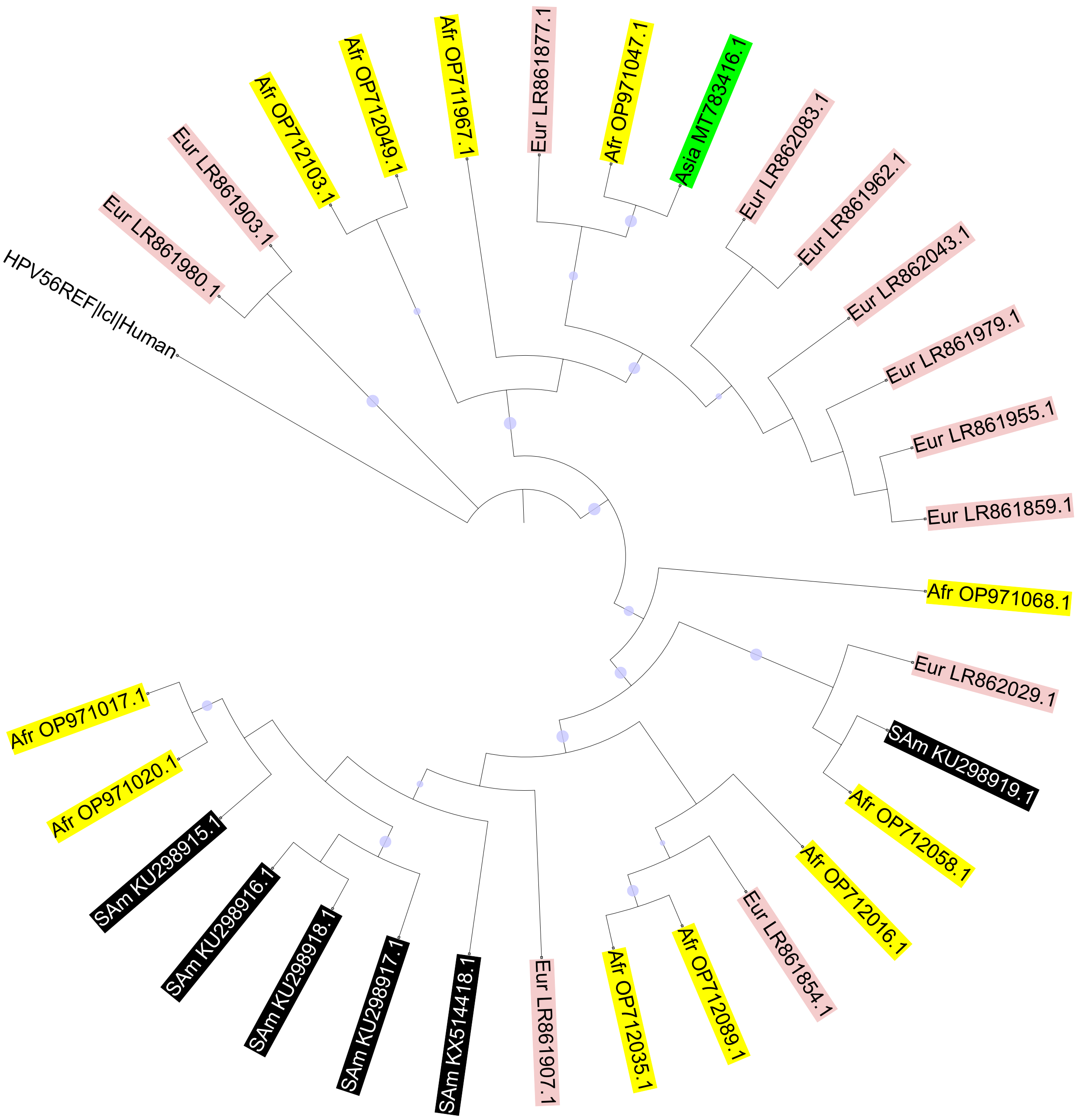
HPV56REF||cl||Human
Eur LR861980.1
Eur LR861903.1
Afr OP712103.1
Afr OP712049.1
Afr OP711967.1
Eur LR861877.1
Afr OP971047.1
Asia MT783416.1
Eur LR862083.1
Eur LR861962.1
Eur LR862043.1
Eur LR861979.1
Eur LR861955.1
Eur LR861859.1
Afr OP971068.1
Eur LR862029.1
SAm KU298919.1
Afr OP712058.1
Afr OP712016.1
Eur LR861854.1
Afr OP712089.1
Afr OP712035.1
Eur LR861907.1
SAm KX514418.1
SAm KU298917.1
SAm KU298918.1
SAm KU298916.1
SAm KU298915.1
Afr OP971020.1
Afr OP971017.1

HPV59

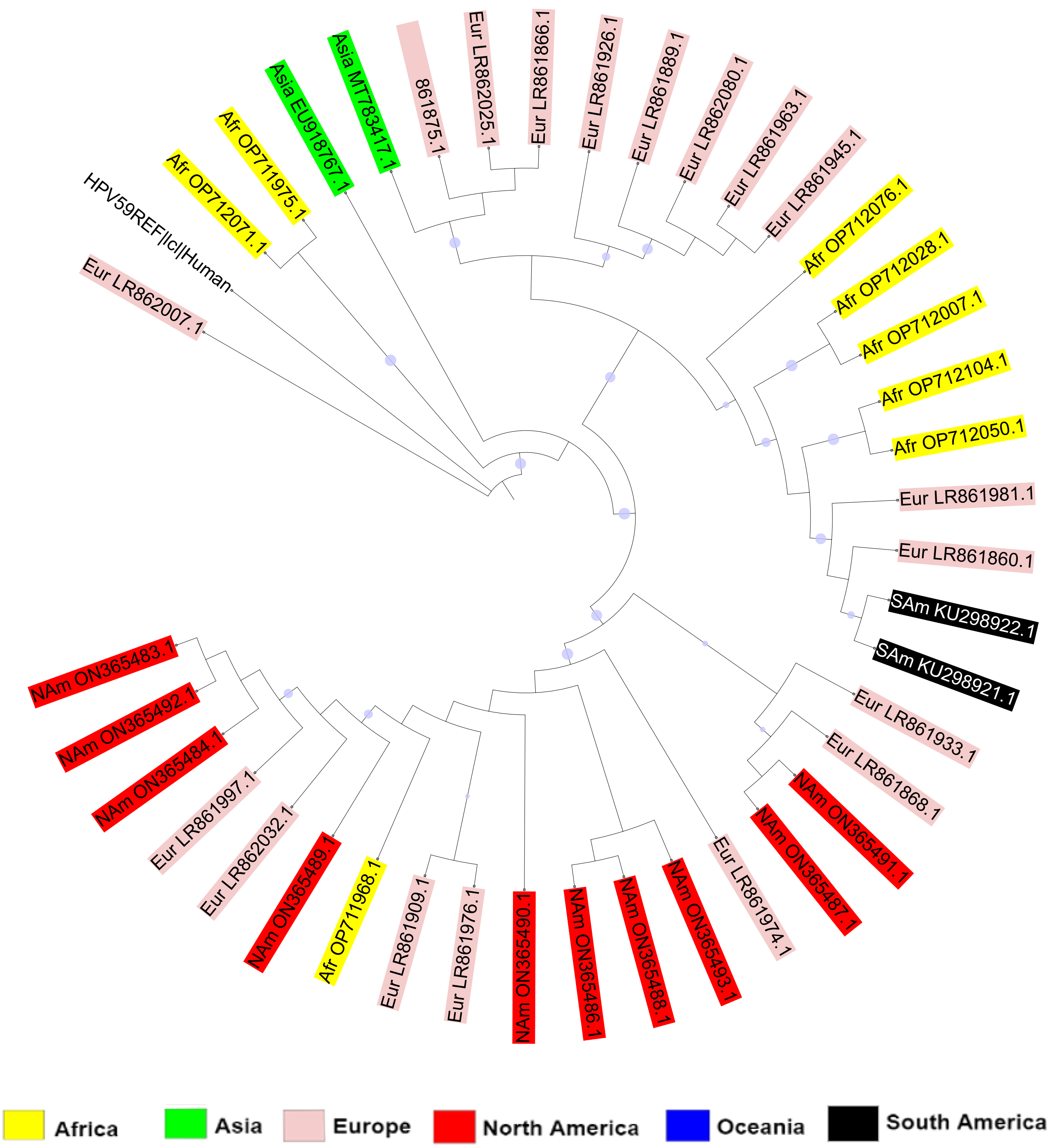

Figure S1. Maximum likelihood tree topologies of HR-HPV isolates from different regions of the world. Multiple sequence alignments were performed using MAFFT v7.525, and phylogenetic analysis was conducted using IQ-TREE2 multicore v2.0.7 with SH-aLRT/UFboot tests for 1000 replicates. Reference genome sequences for each HR-HPV type are included. Purple dots indicate branches with support values ≥70%, with larger dots representing relatively higher support.

2. *Intra-African diversity*

HPV35

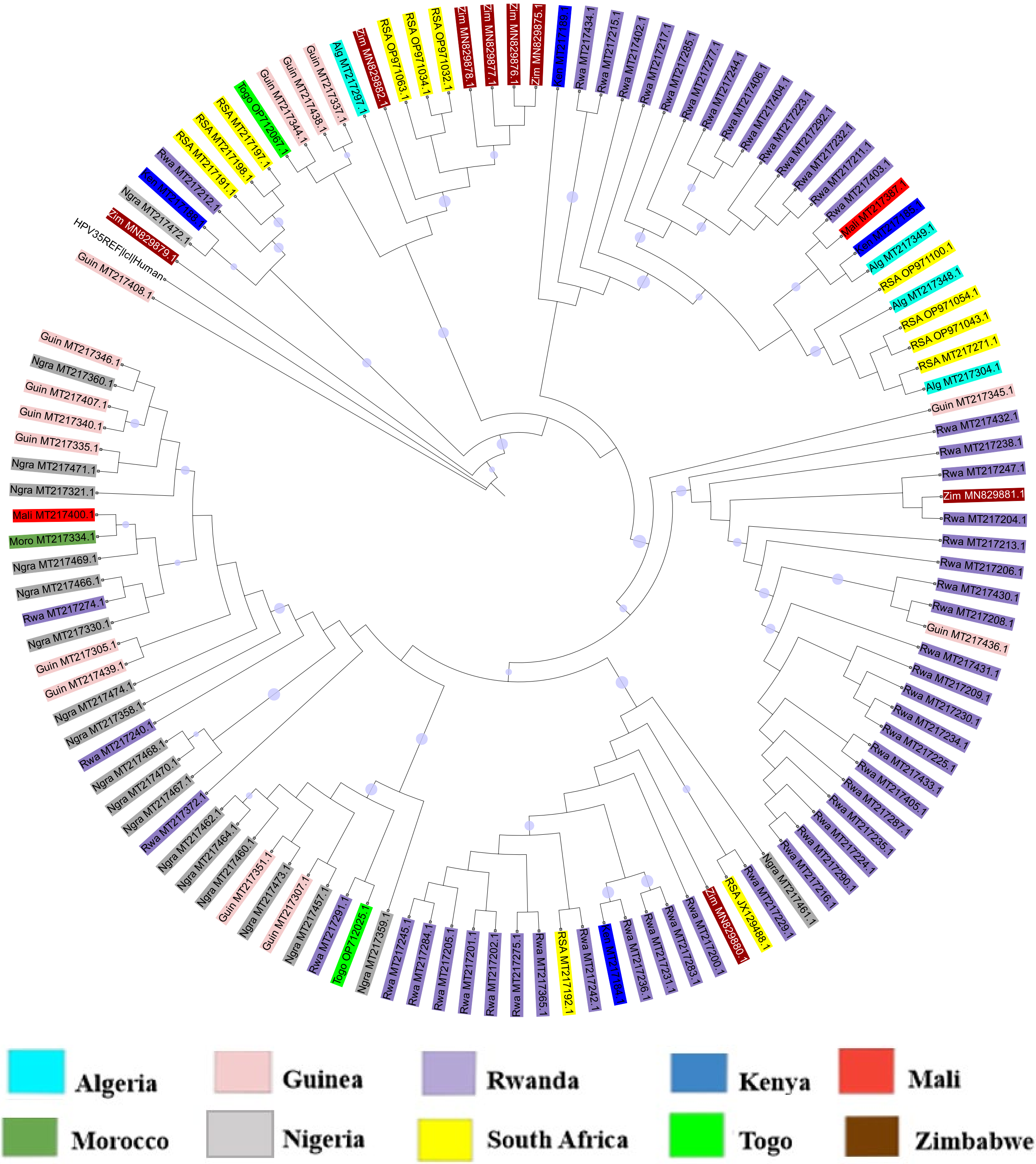

Figure S2. Maximum likelihood tree topologies of complete genomes of HPV35 isolates from countries across Africa. Multiple sequence alignments of HPV35 complete genomes were performed using MAFFT v7.525, and phylogenetic analysis was conducted using IQ-TREE2 multicore v2.0.7 with SH-aLRT/UFboot tests for 1000 replicates. Purple dots indicate branches with support values ≥70%, where larger dots represent relatively higher support compared to other branches on the same tree. Countries represented in this analysis include Algeria (4), Guinea (14), Rwanda (52), Kenya (4), Mali (2), Morocco (1), Nigeria (19), South Africa (12), Togo (2) and Zimbabwe (8).

3. *Commands Used for MAFFT Sequence Alignments*

- mafft world16.fasta > world16_aligned.fasta
- mafft world18.fasta > world18_aligned.fasta
- mafft world35.fasta > world35_aligned.fasta
- mafft world51.fasta > world51_aligned.fasta
- mafft world56.fasta > world56_aligned.fasta
- mafft world59.fasta > world59_aligned.fasta

- mafft Africa16.fasta > world16_aligned.fasta
- mafft Africa18.fasta > world18_aligned.fasta
- mafft Africa35.fasta > world35_aligned.fasta
- mafft Africa51.fasta > world51_aligned.fasta
- mafft Africa56.fasta > world56_aligned.fasta
- mafft Africa59.fasta > world59_aligned.fasta

- mafft world16_cons.fasta > world16_cons_aligned.fasta
- mafft world16_cons.fasta > world16_cons_aligned.fasta
- mafft world16_cons.fasta > world16_cons_aligned.fasta
- mafft world16_cons.fasta > world16_cons_aligned.fasta
- mafft world16_cons.fasta > world16_cons_aligned.fasta
- mafft world16_cons.fasta > world16_cons_aligned.fasta

- mafft Africa 16_cons.fasta > world16_cons_aligned.fasta
- mafft Africa 16_cons.fasta > world16_cons_aligned.fasta
- mafft Africa 16_cons.fasta > world16_cons_aligned.fasta
- mafft Africa 16_cons.fasta > world16_cons_aligned.fasta
- mafft Africa 16_cons.fasta > world16_cons_aligned.fasta
- mafft Africa16_cons.fasta > world16_cons_aligned.fasta

4. *Commands used for construction of phylogenetic trees*

- iqtree2 -s world16_aligned_trimmed.fasta -B 1000 -alrt 1000 -T AUTO
- iqtree2 -s world18_aligned_trimmed.fasta -B 1000 -alrt 1000 -T AUTO
- iqtree2 -s world35_aligned_trimmed.fasta -B 1000 -alrt 1000 -T AUTO
- iqtree2 -s world51_aligned_trimmed.fasta -B 1000 -alrt 1000 -T AUTO
- iqtree2 -s world56_aligned_trimmed.fasta -B 1000 -alrt 1000 -T AUTO
- iqtree2 -s world59_aligned_trimmed.fasta -B 1000 -alrt 1000 -T AUTO

- iqtree2 -s Africa16_aligned_trimmed.fasta -B 1000 -alrt 1000 -T AUTO
- iqtree2 -s Africa18_aligned_trimmed.fasta -B 1000 -alrt 1000 -T AUTO
- iqtree2 -s Africa35_aligned_trimmed.fasta -B 1000 -alrt 1000 -T AUTO
- iqtree2 -s Africa51_aligned_trimmed.fasta -B 1000 -alrt 1000 -T AUTO
- iqtree2 -s Africa56_aligned_trimmed.fasta -B 1000 -alrt 1000 -T AUTO
- iqtree2 -s Africa59_aligned_trimmed.fasta -B 1000 -alrt 1000 -T AUTO

- iqtree2 -s world16_cons_aligned.fasta -B 1000 -alrt 1000 -T AUTO
- iqtree2 -s world18_cons_aligned.fasta -B 1000 -alrt 1000 -T AUTO
- iqtree2 -s world35_cons_aligned.fasta -B 1000 -alrt 1000 -T AUTO
- iqtree2 -s world51_cons_aligned.fasta -B 1000 -alrt 1000 -T AUTO
- iqtree2 -s world56_cons_aligned.fasta -B 1000 -alrt 1000 -T AUTO
- iqtree2 -s world59_cons_aligned.fasta -B 1000 -alrt 1000 -T AUTO

- iqtree2 -s Africa16_cons_aligned.fasta -B 1000 -alrt 1000 -T AUTO
- iqtree2 -s Africa18_cons_aligned.fasta -B 1000 -alrt 1000 -T AUTO
- iqtree2 -s Africa35_cons_aligned.fasta -B 1000 -alrt 1000 -T AUTO
- iqtree2 -s Africa51_cons_aligned.fasta -B 1000 -alrt 1000 -T AUTO
- iqtree2 -s Africa56_cons_aligned.fasta -B 1000 -alrt 1000 -T AUTO
- iqtree2 -s Africa59_cons_aligned.fasta -B 1000 -alrt 1000 -T AUTO

Table S1. Distribution and counts of near-complete (≥80%) genomes of high-risk HPV (HR-HPV) types. The total number of sequences for each HR-HPV type per region is shown, along with the corresponding collection years as recorded in the NCBI Virus database.

HPV	*Region*	*Number of near-complete genomes (≥80%)*	*Collection years*
16	Africa	10	2016 and 2017
	Asia	181	2004, 2006, 2007, 2008, 2009, 2010, 2011, 2012, 2013, 2014, 2016, 2017, 2018, 2019, 2020, 2021, 2022 (others not specified)
	Europe	175	2001, 2003, 2005, 2008, 2009, 2010, 2011, 2014, (others not specified)
	North America	8	Not specified
	Oceania	0	N/A
	South America	20	2007, 2010, 2011, (others not specified)
18	Africa	9	2015, 2016, 2017
	Asia	30	2008, (others not specified)
	Europe	76	2008, 2009, 2010, 2011
	North America	22	2022, 2023, (others not specified)
	Oceania	0	N/A
	South America	2	2016, 2017
35	Africa	118	2008, 2016, 2017, (others not specified)
	Asia	66	Not specified
	Europe	10	2010, (others not specified)
	North America	188	Not specified
	Oceania	5	Not specified
	South America	29	Not specified

51	Africa	11	2016, 2017
	Asia	2	2015, 2016
	Europe	22	Not specified
	North America	2	2022
	Oceania	0	N/A
	South America	6	Not specified
56	Africa	11	2016, 2017
	Asia	1	2016
	Europe	12	Not specified
	North America	0	N/A
	Oceania	0	N/A
	South America	6	Not specified
59	Africa	8	2017
	Asia	2	2007, 2016
	Europe	18	Not specified
	North America	10	Not specified
	Oceania	0	N/A
	South America	2	Not specified
