# Supplementary material for: Genetic Diversity of Selected High-Risk HPV Types Prevalent in Africa and Not Covered by Current Vaccines: A Pooled Sequence Data Analysis
Source: Int J Mol Sci. 2025 Nov 15;26(22):11056. doi: 10.3390/ijms262211056 (PMC12652174; doi:10.3390/ijms262211056)
Supplement: Supplementary file 1 [file ijms-26-11056-s001.zip › Supplementary material_Variant calling analysis.pdf]

Supplementary Table S2. Non-synonymous Variants of the E5 Protein in Africa. Variant calling was performed on E5 gene sequences from African countries, retrieved from the NCBI database.

HPV	***Variant***	***Geographical region***													***Total***	
		**Alg**	**CAR**	**Chad**	**Gui**	**Ken**	**Mali**	**Moro**	**Ngra**	**Cong**	**Rwa**	**RSA**	**Togo**	**Zim**	**n/N**	**%(95%CI)**
16	A7T											1/5	0/6		1/11	9 (0–39)
	I44L											5/5	6/6		11/11	100 (72–100)
	L48V											2/5	0/6		2/11	18 (3–45)
	I65V											4/5	6/6		10/11	91 (59–100)
	I65L											1/5	0/6		1/11	9 (0–38)
18	M14L											3/4	5/6		8/10	80 (44–97)
	V42I											3/4	5/6		8/10	80 (44–97)
	L72S											3/4	2/6		5/10	50 (19–81)
	L72F											0/4	3/6		3/10	30 (7–65)
35	D3N	0/5			1/27	0/8	0/4	0/2	0/28		2/92	0/14	0/2	0/7	3/189	2 (0–5)
	L4I	0/5			0/27	0/8	0/4	0/2	0/28		1/92	0/14	0/2	0/7	1/189	1 (0–3)
	A6G	0/5			1/27	0/8	0/4	0/2	0/28		0/92	0/14	0/2	0/7	1/189	1 (0–3)
	L15V	0/5			0/27	0/8	0/4	0/2	0/28		0/92	0/14	0/2	0/7	1/189	1 (0–3)
	A40S	0/5			0/27	0/8	0/4	1/2*	1/28		0/92	0/14	0/2	0/7	2/189	1 (0–4)
	I42L	0/5			0/27	0/8	0/4	0/2	0/28		1/92	0/14	0/2	0/7	1/189	1 (0–3)
	C60G	0/5			0/27	0/8	0/4	0/2	0/28		1/92	0/14	0/2	0/7	1/189	1 (0–3)
	M70I	0/5			1/27	0/8	0/4	0/2	0/28		0/92	0/14	0/2	0/7	1/189	1 (0–3)
	M72I	0/5			0/27	2/8**	0/4	0/2	0/28		0/92	0/14	0/2	0/7	2/189	1 (0–4)
59	Q24P												7/10		7/10	70 (35–93)
	T32A												7/10		7/10	70 (35–93)
	V41L												7/10		7/10	70 (35–93)
	L51I												7/10		7/10	70 (35–93)
	L63I												7/10		7/10	70 (35–93)

Differences in the geographical distribution of variants were assessed using two-tailed Fisher's exact tests in GraphPad Prism v10.6.1. Variants occurring at <1% frequency were reported descriptively and excluded from statistical testing to avoid over-interpretation. Statistically significant associations were indicated by asterisks ($p < 0.05$ to $p < 0.0001$), highlighting variants disproportionately represented in specific regions. No multiple-testing correction was applied, as the analyses were primarily descriptive and exploratory.

*$p < 0.05$ (two-tailed Fisher test)

**$p < 0.01$ (two-tailed Fisher test)

***$p < 0.001$ (two-tailed Fisher test)

****$p < 0.0001$ (two-tailed Fisher test)

Supplementary Table S3. Non-synonymous Variants of the E6 Protein in Africa. Variant calling was performed on E6 gene sequences from African countries, retrieved from the NCBI database.

HPV	*Variant*	*Geographical region*													*Total*	
		**Alg**	**CAR**	**Chad**	**Gui**	**Ken**	**Mali**	**Moro**	**Ngra**	**Cong**	**Rwa**	**RSA**	**Togo**	**Zim**	**n/N**	**%(95%CI)**
16	R10T											1/5	0/6		1/11	9 (0–38)
	R10I											2/5	6/6		8/11	73(39–94)
	Q14D											3/5	6/6		9/11	82 (48–97))
	Q14H											2/5	0/6		2/11	18 (2–45)
	H78Y											5/5	6/6		11/11	100 (72-100)
	L83V											2/5	0/6		2/11	18 (2-45)
18	N129K								1/2			3/4	4/5		8/11	73 (39–93)
35	E7Q	0/5	0/4	1/4*	0/24	0/9	0/4	0/2	0/25		0/82	0/18	0/2	0/8	1/187	1(0-3)
	E18K	0/5	0/4	0/4	0/24	0/9	0/4	0/2	0/25		1/82	0/18	0/2	0/8	1/187	1 (0-3)
	E20K	0/5	0/4	0/4	1/24	0/9	0/4	0/2	0/25		0/82	0/18	0/2	0/8	1/187	1 (0-3)
	I73V	0/5	0/4	0/4	0/24	0/9	1/4	0/2	0/25		1/82	1/18	0/2	0/8	3/187	2 (0-5)
	W78R	4/5**	1/4	0/4	0/24	2/9	1/4	1/2	0/25*		7/82	5/18*	0/2	1/8	22/187	11 (7-17)
	E86D	0/5	0/4	0/4	0/24	0/9	0/4	0/2	0/25		1/82	0/18	0/2	0/8	1/187	1 (0-3)
	H98Y	0/5	0/4	0/4	0/24	0/9	1/4*	0/2	0/25		0/82	0/18	0/2	0/8	1/187	1 (0-3)
	E121D	0/5	0/4	0/4	0/24	0/9	0/4	0/2	0/25		1/82	0/18	0/2	0/8	1/187	1 (0-3)
	R131Q	0/5	0/4	0/4	0/24	0/9	0/4	0/2	0/25		2/82	0/18	0/2	0/8	2/187	1 (0-4)
	E148K	0/5	0/4	0/4	0/24	0/9	0/4	0/2	0/25		0/82	1/18	0/2	0/8	1/187	1 (0-3)
51	K72R											0/4	2/8		2/12	17 (2-48)
	S100L											0/4	6/8		6/12	50 (21–79)
56	S14R											1/4	3/7		4/11	36 (10–69)
	K54N											1/4	3/7		4/11	36 (10–69)
	D60N											3/4	4/7		7/11	64 (31-89)
59	N50S												3/10		3/10	30 (7–65)

Differences in the geographical distribution of variants were assessed using two-tailed Fisher's exact tests in GraphPad Prism v10.6.1. Variants occurring at <1% frequency were reported descriptively and excluded from statistical testing to avoid over-interpretation. Statistically significant associations were indicated by asterisks ($p < 0.05$ to $p < 0.0001$), highlighting variants disproportionately represented in specific regions. No multiple-testing correction was applied, as the analyses were primarily descriptive and exploratory.

*p < 0.05 (two-tailed Fisher test)

**p < 0.01 (two-tailed Fisher test)

***p < 0.001 (two-tailed Fisher test)

****p < 0.0001 (two-tailed Fisher test)

Supplementary Table S4. Non-synonymous Variants of the E7 Protein in Africa. Variant calling was performed on E7 gene sequences from African countries, retrieved from the NCBI database.

HPV	*Variant*	*Geographical region*													*Total*	
		**Alg**	**CAR**	**Chad**	**Gui**	**Ken**	**Mali**	**Moro**	**Ngra**	**Cong**	**Rwa**	**RSA**	**Togo**	**Zim**	**n/N**	**%(95%CI)**
16	N29S									1/2		2/5	6/6		9/13	69 (39-91)
	S63C									0/2		1/5	0/6		1/13	8 (0-36)
18	H2Y											3/4	4/5		7/9	78 (40-97)
	N92S											3/4	2/5		5/9	56 (21-86)
	N92K											0/4	2/5		2/9	22 (3–60)
35	Y23H	0/5			3/26	2/10	0/4	0/2	0/27*		17/94*	1/18	0/2	0/8	23/196	12 (8– 17)
	G41C	0/5			0/26	0/10	0/4	0/2	0/27		0/94	0/18	0/2	2/8**	2/196	1 (0–4)
	S51C	0/5			0/26	0/10	0/4	0/2	1/27		0/94	0/18	0/2	0/8	1/196	1 (0-3)
	E63K	1/5			3/26	0/10	0/4	0/2	7/27		20/94	0/18*	1/2	0/8	32/196	16 (11-22)
51	K9Q											0/4	2/8		2/12	17(2– 48)
	G77E											0/4	6/8		6/12	50 (21-79)
	T79N											0/4	6/8		6/12	50 (21-79)
56	D10E											3/4	4/7		7/11	64 (31-89)
	V12I											3/4	7/7		10/11	91 (58-100)
	E67K											0/4	1/7		1/11	9 (0– 40)
	E67Q											4/4	3/7		7/11	64 (31-89)
	Q77H											1/4	3/7		4/11	36 (10 -69)
59	H2Y												1/10		1/10	10 (0–45)
	Q18H												7/10		7/10	70 (35–93)
	Y20F												7/10		7/10	70 (35–93)
	T95A												7/10		7/10	70 (35–93)

Differences in the geographical distribution of variants were assessed using two-tailed Fisher's exact tests in GraphPad Prism v10.6.1. Variants occurring at <1% frequency were reported descriptively and excluded from statistical testing to avoid over-interpretation. Statistically significant associations were indicated by asterisks ($p < 0.05$ to $p < 0.0001$), highlighting variants disproportionately represented in specific regions. No multiple-testing correction was applied, as the analyses were primarily descriptive and exploratory.

*$p < 0.05$ (two-tailed Fisher test)

**$p < 0.01$ (two-tailed Fisher test)

***$p < 0.001$ (two-tailed Fisher test)

****$p < 0.0001$ (two-tailed Fisher test)

Supplementary Table S5. Non-synonymous Variants of the L1 Protein in Africa. Variant calling was performed on L1 gene sequences from African countries, retrieved from the NCBI database.

HPV	*Variant*	*Geographical region*													*Total*	
		**Alg**	**CAR**	**Chad**	**Gui**	**Ken**	**Mali**	**Moro**	**Ngra**	**Cong**	**Rwa**	**RSA**	**Togo**	**Zim**	**n/N**	**% (95%CI)**
16	H76Y											5/6	7/7		12/13	92 (64– 100)
	T176N											5/6	7/7		12/13	92 (64– 100)
	A179T											2/6	0/7		2/13	15 (2– 45)
	N181T											3/6	0/7		3/13	23 (5– 54)
	T266A											6/6	7/7		13/13	100 (75–100)
	S282P											2/6*	7/7*		9/13	69 (39– 91)
	T353P											4/6	6/7		10/13	77 (46-95)
	S396A											0/6	2/7		2/13	15 (2–45)
	L474F											5/6	7/7		12/13	92 (64-100)
18	L3M											0/4	1/7		1//11	9(0– 40)
	T88N											3/4	7/7		10/11	91 (58– 100)
	Q273P											0/4	1/7		1/11	9(0 – 40)
	V323I											3/4	5/7		8/11	73 (39– 94)
	S495F											1/4	0/7		1/11	9 (0– 40)
35	Q55P	0/6			0/27	0/10	0/4	0/2	0/28		1/86	0/18	0/2	0/8	1/191	1 (0-3)
	F73C	0/6			0/27	0/10	0/4	0/2	0/28		0/86	1/18	0/2	0/8	1/191	1 (0-3)
	H120Y	0/6			0/27	0/10	0/4	0/2	0/28		1/86	0/18	0/2	0/8	1/191	1 (0-3)
	A182P	1/6			3/27	1/10	0/4	0/2	0/28		2/86**	4/18	1/2	5/8***	17/191	9 (5 – 14)
	K278T	0/6			0/27	0/10	0/4	0/2	0/28		1/86	0/18	0/2	0/8	1/191	1 (0-3)
	S348T	4/6*			2/27*	3/10	1/4	1/2	2/28*		25/86	8/18*	0/2	0/8	46/191	24 (18-31)
	S349T	0/6			2/27	0/10	0/4	0/2	0/28		16/86*****	0/18	0/2	0/8	18/191	9 (6 – 14)
	P438A	0/6			0/27	0/10	0/4	0/2	0/28		1/86	0/18	0/2	0/8	1/191	1 (0-3)
	S494F	0/6			0/27	0/10	0/4	0/2	1/28		0/86	0/18	0/2	0/8	1/191	1 (0-3)
	K501Q	0/6			1/27	0/10	0/4	0/2	0/28		0/86	0/18	0/2	0/8	1/191	1 (0-3)
51	P178A											0/4**	9/9**		9/13	69 (39-91)
	V264G											4/4	8/9		12/13	92 (64 -100)
	G265S											4/4	8/9		12/13	92 (64 -100)
	D273N											0/4	6/9		6/13	46 (19-75)
	T354P											0/4	2/9		2/13	15 (2 –45)
	A436D											0/4*	7/9*		7/13	54 (25-81)
	R479K											0/4*	7/9*		7/13	54 (25-81)
56	N91D											1/4	0/7		1/11	9 (0–40)

	I190V	2/4	0/7	2/11	18 (2–52)
	R356Q	0/4	2/7	2/11	18 (2–52)
	K475R	1/4	3/7	4/11	36 (12-68)
59	E26D		7/10	7/10	70 (35–93)
	R71K		1/10	1/10	10 (0–45)
	K76N		7/10	7/10	70 (35–93)
	L122F		7/10	7/10	70 (35–93)
	T140N		7/10	7/10	70 (35–93)
	P177S		1/10	1/10	10 (0–45)
	T178N		7/10	7/10	70 (35–93)
	V180A		1/10	1/10	10 (0–45)
	N216T		7/10	7/10	70 (35–93)
	L271I		7/10	7/10	70 (35–93)
	N285T		3/10	3/10	30 (7–65)
	S351P		5/10	5/10	50 (19–81)
	V355E		1/10	1/10	10 (0–45)
	S360N		1/10	1/10	10 (0–45)
	P449T		2/10	2/10	20 (3–56)
	P449S		4/10	4/10	40 (12–74)
	D451N		1/10	1/10	10 (0–45)
	S496A		1/10	1/10	10 (0–45)
	R507K		2/10	2/10	20 (3–56)

Differences in the geographical distribution of variants were assessed using two-tailed Fisher's exact tests in GraphPad Prism v10.6.1. Variants occurring at <1% frequency were reported descriptively and excluded from statistical testing to avoid over-interpretation. Statistically significant associations were indicated by asterisks ($p < 0.05$ to $p < 0.0001$), highlighting variants disproportionately represented in specific regions. No multiple-testing correction was applied, as the analyses were primarily descriptive and exploratory.

*$p < 0.05$ (two-tailed Fisher test)

**$p < 0.01$ (two-tailed Fisher test)

***$p < 0.001$ (two-tailed Fisher test)

****$p < 0.0001$ (two-tailed Fisher test)
